# Biology, care, and outcomes of gestational breast cancers: a review

**DOI:** 10.1007/s10549-025-07684-9

**Published:** 2025-03-28

**Authors:** Niharika Duggirala, Shiliang Zhang, Aashini Master, Rashmi Rao, Nimmi S. Kapoor, Aditya Bardia, Marla Lipsyc-Sharf

**Affiliations:** David Geffen School of Medicine at University of California Los Angeles, Los Angeles, CA USA

**Keywords:** Breast cancer, Gestational, Pregnancy, Premenopausal, Young women

## Abstract

**Purpose:**

The incidence of gestational breast cancers, breast cancers diagnosed during pregnancy, is increasing. There is a critical need to understand the pathophysiology, treatment recommendations, and remaining questions regarding care and therapeutics for this complex condition.

**Methods:**

Here, we review existing data regarding evaluation and management of gestational breast cancer, including safe imaging modalities, timing and choice of chemotherapy, evidence regarding targeted therapies during pregnancy. We highlight the importance of multidisciplinary care including oncologic, obstetric, and psychosocial care.

**Results:**

Gestational breast cancers are associated with unique biologic and clinicopathologic features that are impacted by physiologic changes of pregnancy such as upregulation of target genes associated with cell proliferation and immune regulation. Patients with gestational breast cancers more often present at advanced stages, are more likely to have aggressive tumor subtypes (i.e., triple negative or HER2 positive), and overall have worse prognoses than patients with non-gestational breast cancers. In this review, we synthesize recommendations for treatment strategies based on pregnancy trimester, optimal timing and choice of surgery, chemotherapy, targeted therapies, and psychosocial support.

**Conclusion:**

Developing a framework for clinical care and treatment of patients with gestational breast cancers is integral to improving outcomes for patients with gestational breast cancers. Optimal treatment includes collaborative management with a multidisciplinary team dedicated to both maternal and fetal care.

## Introduction

Breast cancer is the most frequently diagnosed cancer among women aged 20–39 in the United States, and its incidence has been rising steadily since 2010 [1]. With increased breast cancer diagnoses in young women coupled with increases in maternal age in recent decades, more pregnant women are being diagnosed with breast cancer than ever before. Breast cancer diagnosed during pregnancy, gestational breast cancer, is relatively rare. Current estimates indicate that breast cancer complicates approximately 1 in 3000 pregnancies [2]. However, among all young women with breast cancer, this is not uncommon: about 20% (1 in 5) breast cancers diagnosed in women aged 25–29 occur either during gestation or within the first postpartum year [3].

The increasing incidence of pregnancy-associated breast cancers (which includes breast cancers diagnosed during gestation and up to 5–10-year postpartum) presents unique medical, psychological, and sociological challenges. In the diagnosis and treatment of gestational breast cancer, maximally effective treatment is balanced with maternal and fetal safety. Additionally, women with gestational cancer often endure considerable psychosocial stress due to many factors, including anxiety regarding their health, their baby’s health, caretaking, and future family planning. Here, we aim to review the biology, care, and outcomes of breast cancer diagnosed and treated during pregnancy. We seek to offer a practical framework for clinicians to effectively evaluate and manage this important and increasingly common disease.

## Biology and pathologic features of gestational breast cancers

Breast cancer diagnosed during pregnancy is associated with multiple known high-risk features such as young age at diagnosis, large tumor size, high tumor grade, high frequency of lymph node involvement, and higher-risk tumor subtypes (i.e., triple-negative and HER2-positive breast cancers) [4, 5]. Histologically, gestational breast cancers are similar to non-gestational. The majority are invasive ductal carcinomas, with a smaller proportion presenting as other histologies, such as lobular carcinoma. Regarding tumor subtype, existing data suggest that most patients diagnosed with breast cancer during pregnancy have tumors that are hormone receptor negative. While HER2-positive gestational breast cancers may be more common than hormone receptor-positive tumors in this population, still the majority seem to be HER2 negative [4].

Physiologic changes during pregnancy including hormonal upregulation, cellular proliferation, and alterations in the immune system may contribute to the pathogenesis of gestational breast cancer. Breast cancers diagnosed during pregnancy have been associated with increased expression of multiple hormone target and cell proliferation genes including AURKA, BIRC5, MKI6, and MMP11. Additionally, increased maternal immune tolerance facilitates safety of fetal growth which may also contribute to reduced immune surveillance in recognizing malignancies. Whole-genome sequencing analyses have demonstrated that compared to non-gestational tumors, epithelial cells from breast cancers diagnosed during pregnancy have higher expression of genes that regulate immune and inflammatory responses (e.g., PD1, PD-L1), DNA repair (e.g., BRCA1/2, FEN1, Sig20), cell proliferation (e.g., IGF1 and B-catenin), and pro-oncogenes (e.g., MYC, SRC, FOS) [6, 7].

## Clinical presentation and risk factors

**Fig. 1:**
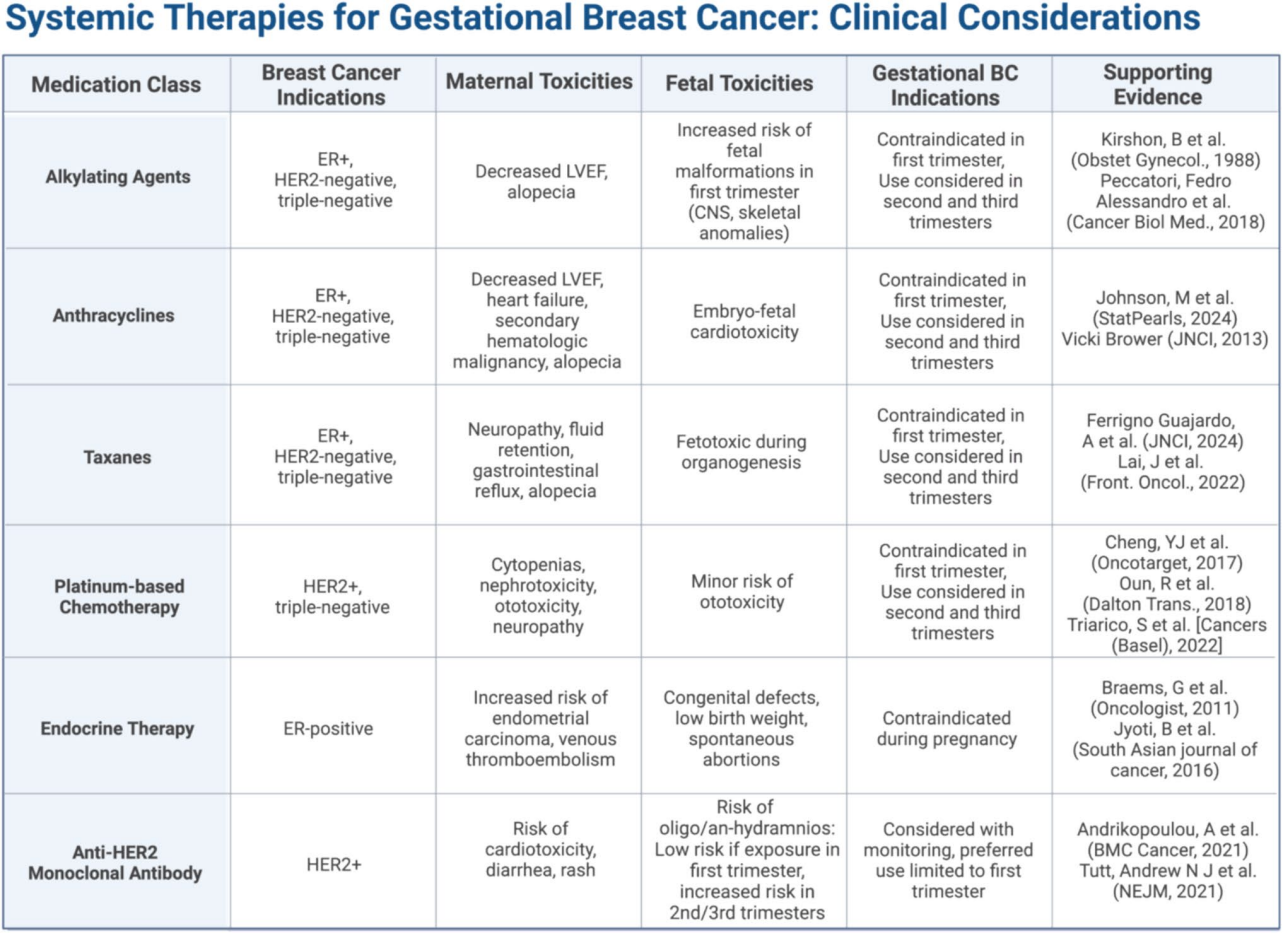
Indications, contraindications, and clinical considerations for administration of systemic therapy in gestational breast cancer. BC, breast cancer; CNS, central nervous system; ER, estrogen receptor; HER2, human epidermal growth factor receptor 2; LVEF, left ventricular ejection fraction. Created in BioRender. Lipsyc-sharf, M. (2025) https://BioRender.com/m87n897

As the incidence of gestational breast cancer rises, emerging data are providing insights into its risk factors, presentation, and clinical features, highlighting how these may differ from those seen in non-gestational breast cancer patients (Fig. 1).

### Risk factors

Risk factors for gestational breast cancer appear to be similar to risk factors for non-gestational breast cancers (Table 1). In particular, family history of breast cancer, age over 30 at first pregnancy, early menarche, lack of previous breastfeeding, and increased BMI have all been associated with a higher risk of breast cancer[1]. Additionally, there are multiple pathogenic germline mutations associated with increased lifetime risk of breast cancer. In particular, women with germline *BRCA1* or *BRCA2* mutation carriers are at an increased likelihood of developing breast cancer in their lifetime, with a risk exceeding 70% [8]. The average age at diagnosis for *BRCA1* carriers is between 41 and 50 years [9], highlighting the need for high-risk breast cancer screening, including the consideration of early screening, and the addition of breast MRI in this population. Appropriate preconception counseling should be provided for *BRCA* mutation carriers who choose to become pregnant, along with regular physical examinations and imaging before, during, and after pregnancy, to monitor their breast cancer risk effectively [10].

**Table 1:** Clinicopathologic features of gestational versus non-gestational breast cancers

	Gestational	Non-gestational
Risk factors**	Pathogenic germline mutations (i.e., BRCA1, BRCA2, and others)	
	Starting menstrual periods before age 12	
	Personal history of malignant or benign breast tumors	
	Age	
	Family history of breast cancer (BC)	
	Previous radiation to chest/breasts (i.e., for treatment of lymphoma)	
	Sedentary lifestyle	
	Obesity	
	Age at first live birth more than 30 years old	
	Drinking alcohol	
Presentation	Typically presents with a patient- or provider-detected breast mass. Rarely diagnosed via screening mammogram	Many (~ 40%) are diagnosed via screening mammogram in asymptomatic patients, while the remainder are patient- or provider detected
Breast imaging	Ultrasound is the first-line imaging tool	Mammography and ultrasound are first-line imaging tools
	Mammography used safely with US is insufficient	If needed for further evaluation, a breast MRI with contrast can be done
	Non-contrast MRI may be considered for an individual patient pending US and mammogram	
	Contrast-enhanced MRI is contraindicated	
Staging scans	Ultrasound of the abdomen and chest-X-rays with fetal shielding can be used	CT chest/abdomen/pelvis and bone scan OR PET/CT scans
	If additional imaging is needed, whole-body diffusion-weighted MRI can be used for staging for gestational BCs	
Breast Surgery	Generally, it is recommended to wait to pursue surgery until after the first trimester	Patients and their surgical oncologists mutually decide regarding choice between mastectomy and breast-conserving surgery
	Often, mastectomy is chosen instead of breast-conserving surgery (BCS) because radiation is contraindicated during pregnancy (see below)	Sentinel lymph node evaluation is performed as per standard of care
	Use of isosulfan blue for sentinel lymph node evaluation is contraindicated due to theoretical risk of maternal anaphylaxis	
Systemic therapy considerations*	Systemic therapy is chosen based on factors, such as subtype of breast cancer and gestational age	Standard treatment is indicated (i.e., with chemotherapy, immunotherapy, endocrine therapies, and targeted therapies)
	Endocrine therapy and targeted therapies are contraindicated	
	Supportive care medications should be optimized to balance maternal support and fetal risk	
Radiation therapy	Radiation is contraindicated during pregnancy	Adjuvant radiation is often utilized after breast-conserving surgery or after mastectomy if there are high-risk features
Other considerations	Pre-chemotherapy fertility/oocyte preservation is deferred as this is not pursued during pregnancy	For pre-menopausal patients, consider fertility preservation/oocyte cryopreservation prior to chemotherapy
	Pathologic assessment of placental tissue is recommended following delivery	

US, ultrasound; BC, breast cancer

^*^see Fig. 1

^**^Known risk factors appear to be similar in both gestational and non-gestational breast cancers

### Clinical presentation

**Fig. 2:**
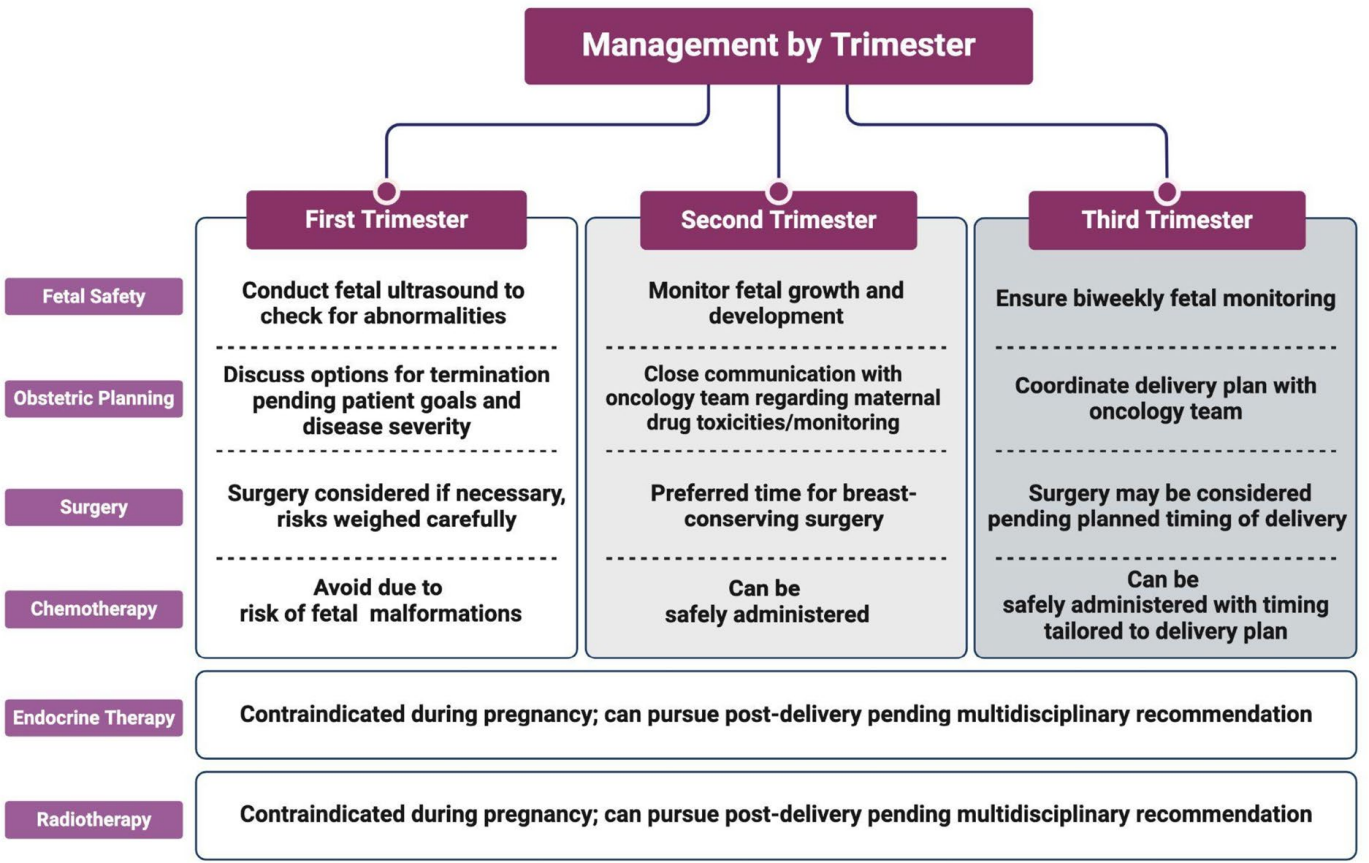
Management of gestational breast cancer by trimester. It defines a trimester-based approach to the treatment of gestational breast cancer, including timing of obstetric and therapeutic interventions. Created in BioRender. Lipsyc-sharf, M. (2025) https://BioRender.com/v24a956

In the general population, approximately 40% of breast cancers [11] are detected through mammography. However, mammographically detected breast cancers are rare in pregnant patients with breast cancer. Per current USPSTF guidelines, it is recommended that women begin breast cancer screening through mammography at age 40, well beyond the average maternal age during pregnancy. Additionally, even for women aged 40 and older, screening mammography is not routinely performed during pregnancy, and most commonly pregnant women elect to postpone mammography until after delivery. As a result, gestational breast cancers typically present with a patient- or provider-detected breast mass [12] (Fig. 2).

During the first trimester of pregnancy, breast tissue undergoes significant physiological changes driven by fluctuations in hormones, such as estrogen, progesterone, and prolactin. Specifically, there is increased proliferation of blood vessels and lobules within the breast tissue [11]. These physiological changes can obscure the presentation of breast cancer, as normal pregnancy-related changes such as increased breast density, nodularity, skin changes, and nipple discharge may mask malignancy [10]. Consequently, distinguishing normal breast tissue changes from pathological findings can be challenging through at-home breast exams and palpation, leading to delays in diagnosis. These delays average 5–7 months, which often results in tumor progression during pregnancy and a more advanced stage at diagnosis [10].

## Diagnosis and evaluation

Once a breast mass is identified, diagnosis and staging of breast cancer during pregnancy is similar to those in non-pregnant individuals but requires modifications as necessary to prioritize fetal safety. In non-pregnant patients, mammography, ultrasound, and, for some, breast MRI are used to evaluate breast masses. For pregnant women, however, ultrasound remains the first-line imaging tool for evaluating breast masses due to its safety and high sensitivity and negative predictive value, making it effective in distinguishing between benign and malignant tissue changes [13]. Mammography can be safely used in pregnancy with minimal radiation dose delivered to the fetus and should be used when ultrasound alone is insufficient for diagnosis. Non-contrast MRI may be considered secondarily if it provides diagnostic benefit and is used in accordance with American College of Radiology guidelines [14]. However, contrast-enhanced MRIs, which are typically utilized in non-gestational breast cancers, are avoided due to the risk of gadolinium crossing the placenta and fetal toxicity [15].

**Fig. 3:**
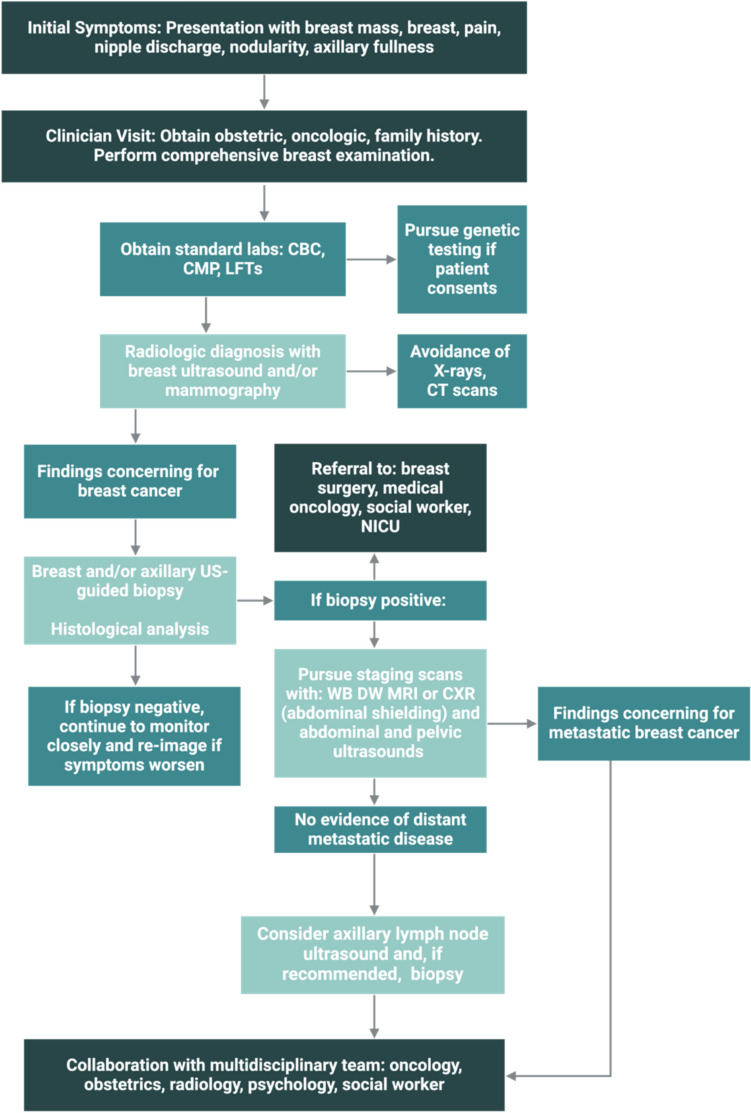
Flowchart of diagnostic and treatment pathway for gestational breast cancer. It illustrates process of diagnostic workup and key clinical decisions. CBC, complete blood count; CMP, comprehensive metabolic panel, LFT, liver function test; NICU, neonatal intensive care unit; WB DW MRI, whole-body diffusion-weighted MRI. Created in BioRender. Lipsyc-sharf, M. (2025) https://BioRender.com/r88p599

If imaging findings are suspicious, biopsy is recommended to obtain a tissue sample for pathologic diagnosis [16]. Once pathologic diagnosis of breast cancer is confirmed, staging is recommended with staging scans. Staging imaging studies should be performed while taking precautions to minimize radiation exposure to the fetus. Ultrasound is recommended for visualizing and assessing the abdomen and pelvis, while chest X-rays with fetal shielding can be used for evaluating the chest [6]. Alternatively, whole-body diffusion-weighted MRI may be used for staging scans during pregnancy [17]. It is generally recommended that abdominal X-ray and systemic CT scans are avoided unless absolutely necessary during pregnancy to limit exposure to ionizing radiation. The risk to a fetus from ionizing radiation is dependent on the gestational age at the time of exposure and the dose of radiation. Fetal risk of anomalies, growth restriction, or abortion have not been reported with radiation exposure of less than 50 mGy, a level above the range of exposure for diagnostic procedures [18]. However, if possible, ultrasound, chest X-rays, and diffusion-weighted MRIs are preferred such that ionizing radiation may be avoided [19] (Fig. 3).

## Treatment

Optimizing outcomes for patients with gestational breast cancer requires treatment strategies that closely align with those recommended for non-pregnant patients, while being adapted to minimize harm to the fetus and effectively treat the malignancy. Treatment plans are adjusted depending on the trimester of pregnancy and careful consideration of maternal and fetal well-being.

### Early-stage breast cancer

In general, early-stage breast cancer is treated with both local therapy (i.e., surgery with or without radiation) and systemic therapy (i.e., medications). While breast cancer surgery (including breast-conserving surgery and mastectomy) is considered safe in each trimester of pregnancy, ideally this should be delayed until after gestational age of 12 weeks to optimize outcomes and minimize the risk of a spontaneous miscarriage [20, 21]. A multidisciplinary discussion among breast surgeons, anesthesiologists, and obstetricians can optimize concerns regarding hypoxia, hypotension, hypoglycemia, fever, pain, thrombosis, and infection. After discussion considering the patient’s wishes, the use of fetal heart rate monitoring during surgery to detect fetal distress and the option of betamethasone or dexamethasone for fetal lung maturity should be offered to the patient following institutional guidelines. Anesthetic agents such as propofol do cross the placenta; however, studies indicate that this risk is generally low at clinically recommended doses, and the maternal benefits of surgery usually outweigh the potential fetal harm [22]. Anesthesia can be carried out safely provided due allowance is made for the physiologic changes of pregnancy, such as increase in circulatory volume, postural hypotension, and delayed gastric emptying. The use of post-operative tocometry can be used to identify uterine activity that may be masked by analgesia and thromboprophylaxis with low-molecular weight heparin is indicated in the post-operative state, given that both pregnancy and cancer increase the risk for venous thromboembolism.

**Fig. 4:**
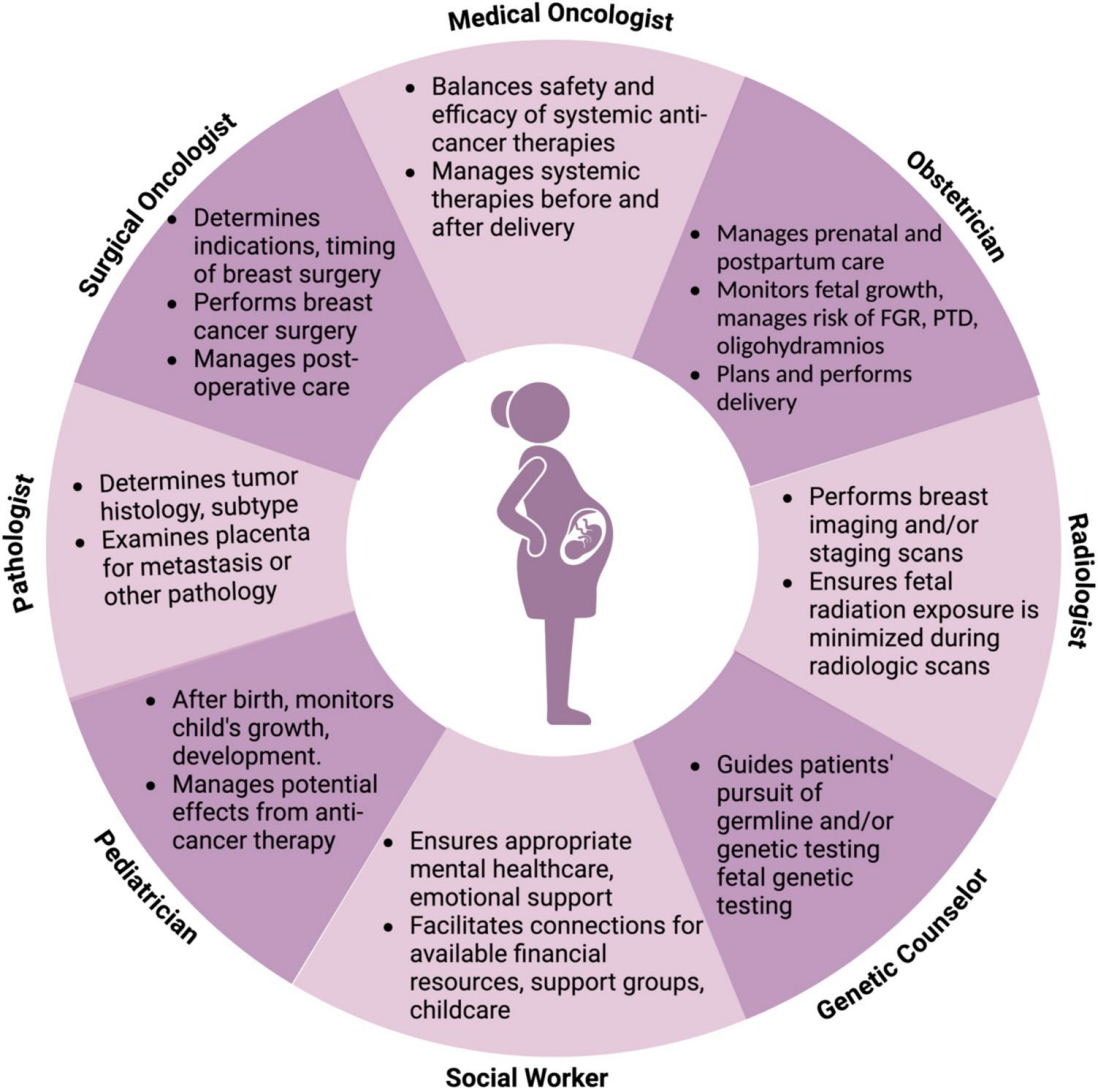
Multidisciplinary care wheel depicting care team involved in the management of gestational breast cancer. It depicts collaborations of various healthcare professionals and their roles in treatment of mother and baby. FGR, fetal growth restriction; PTD, preterm delivery. Created in BioRender. Lipsyc-sharf, M. (2025) https://BioRender.com/r88p599

Since radiation is contraindicated in pregnancy, surgical treatment of choice in the first and early second trimester of pregnancy is often mastectomy unless chemotherapy is also planned to allow for postponement of radiation therapy until after delivery. Otherwise, breast-conserving surgery in the late second and third trimesters is feasible as radiation can be safely postponed until after delivery. In clinically node-negative women, sentinel lymph node biopsy with technetium sulfur colloid for mapping is considered safe; however, use of isosulfan blue dye is contraindicated due to theoretical risk of maternal anaphylaxis [23]. In women with clinically node-positive disease, standard axillary dissection is recommended. While many young patients will have genetic susceptibility or family history of breast cancer, bilateral mastectomy, even with implant-based reconstruction, has been demonstrated to be safe during pregnancy and our group has found that some of these women may choose to undergo prophylactic surgery within a year of delivery even after the index lesion has been removed [24, 25] (Fig. 4).

Radiation therapy presents risks to fetal safety due to exposure to high doses of radiation, which increase the likelihood of fetal malformations, developmental defects, and childhood cancers. For in utero exposure, a radiation dose of less than 0.2 Gy is generally accepted as the fetal safety threshold [12]. Standard chest radiotherapy exposes the mother to up to 60 Gy of radiation, and fetal exposure can reach 2 Gy [26], 10 times greater than the safety range. As such, it is recommended that pregnant patients delay radiation therapy until after delivery.

Gestational age and pregnancy physiology are important considerations when considering systemic therapy during pregnancy. Normal changes in pregnancy such as an increased plasma volume, hepatic oxidations, increased renal clearance, and the third space created by amniotic fluid can affect drug distribution, metabolism, and excretion. Despite these changes during pregnancy, pregnant women receive similar weight-based doses as women who are not pregnant, adjusted with the continuing weight gain [27, 28]. Systemic therapy, particularly chemotherapy is often not recommended during the first trimester due to the elevated risk of spontaneous abortion and potential interference with fetal organogenesis. During the first trimester, chemotherapy is associated with an overall 14% incidence of fetal malformations, with some drugs, such as 5-fluorouracil, having a risk as high as 31% [6, 29]. Specifically, anthracyclines such as doxorubicin and epirubicin are known to induce embryo-fetal cardiotoxicity if given prior to week 13 gestation, as fetal cardiac tissue is vulnerable to chemotherapy-induced damage [30]. Alkylating chemotherapeutic agents such as cyclophosphamide are associated with fetal central nervous system and skeletal anomalies including cleft palate, absent thumbs and eye abnormalities [31]. Additionally, carboplatin chemotherapy, which is included in the treatment of many HER2-positive and triple-negative breast cancers, is thought to be safe to administer in the second and third trimesters with a more favorable toxicity profile compared to other platinum-based chemotherapy, such as cisplatin [32, 33]. Although paclitaxel and docetaxel are fetotoxic during organogenesis [34], recent findings suggest that taxane administration beyond the first trimester is relatively safe, with a 2024 cohort study indicating a 2.2% rate of congenital malformations similar to the general population [35]. These data support the use of regimens containing anthracycline and taxane when treating gestational breast cancer in the second and third trimesters.

While formal studies have not reviewed the differing side-effect profiles of chemotherapy in the pregnant population compared to the non-pregnant population, chemotherapy side effects seem to be less pronounced during pregnancy, and systemic therapy is generally very well tolerated. Supportive care for side effects from chemotherapeutics should follow the general principles regarding medication use in pregnancy. Drugs should not be withheld if necessary and should not be used unless indicated. Studies for use of growth factors for white and red blood cells during pregnancy are overall reassuring and can be used if necessary [36, 37]. There are limited safety data for the use of neurokinin-1 inhibitors; however, diphenhydramine and ondansetron are routinely used and considered acceptable alternatives for anti-emetics in pregnancy. For pre-medication with steroids, methylprednisolone and hydrocortisone are preferred over dexamethasone and betamethasone given the former are extensively metabolized in the placenta [38].

While endocrine therapy, with agents such as tamoxifen and aromatase inhibitors, is commonly used in the treatment of hormone receptor-positive breast cancer, this is contraindicated during pregnancy due to potential teratogenic effects and interaction with fetal tissue [39]. Tamoxifen has been associated with severe congenital defects, low birth weight, and spontaneous abortions in animal studies. A study examining the effects of tamoxifen during any trimester of pregnancy identified 16 cases of live births with congenital anomalies and 122 live births without anomalies, suggesting a high frequency of congenital malformations among pregnancies exposed to tamoxifen [40]. Tamoxifen is therefore contraindicated throughout pregnancy and in the case of in utero exposure, careful follow-up and examination are recommended. Aromatase inhibitors are similarly contraindicated during pregnancy, as they may cause loss of pregnancy or fetal abnormalities including skeletal anomalies, abnormal head morphology, and genital tract anomalies throughout the pregnancy [41].

Previous literature has advised against the use of the trastuzumab anti-HER2 monoclonal antibody, a standard adjuvant agent in HER2-positive breast cancer treatments, in pregnancy due to risk of oligohydramnios or anhydramnios. However, a 2021 review found that less than 20% of cases where trastuzumab was solely administered during the first trimester were complicated by oligo or anhydramnios, compared to over 70% in cases where trastuzumab treatment continued into the second or third trimesters [42]. Regarding fetal outcomes, the same study found 75% of pregnancies in which fetal exposure was limited to the first trimester resulted in live births without congenital abnormalities, as opposed to only 41.7% when treatment with trastuzumab was extended to the second or third trimesters. Overall, fetal exposure to trastuzumab during the first trimester is considered to have relatively low risk, due to known minimal transfer of immunoglobulin IgGs across the placenta in the first trimester [43]. While it is generally recommended that treatment with trastuzumab be delayed until after delivery for optimal fetal protection, given these findings, trastuzumab administration during the first trimester may be considered with close maternal and fetal monitoring.

The use of targeted therapy was previously limited to treatment of metastatic breast cancer, though many of these therapies have recently been introduced into moved to treatment of early-stage breast cancer as well. For example, PARP inhibitors are now used in breast cancer patients with germline *BRCA1/2* mutations both in metastatic and early-stage HER2-negative breast cancers [44]. However, these agents have been associated with embryo-fetal toxicity; further research in this area is required to determine the exact teratogenic profile [45]. Other targeted therapies include CDK4/6 inhibitors, immunotherapy, and trastuzumab emtansine. Preclinical animal studies suggest that use of CDK 4/6 inhibitors during pregnancy may result in decreased fetal growth and/or cardiovascular and skeletal malformations [46]. Given this risk of congenital fetal abnormalities, use of CDK 4/6 inhibitors during pregnancy is not currently recommended. Immunotherapy is also not recommended during pregnancy. While evidence is sparse, existing data suggest that treatment with immune checkpoint inhibitors may be associated with fetal growth restriction during the second and third trimesters [47]. As targeted therapies are used more frequently, further research on the safety and efficacy of targeted therapies during gestational breast cancer is critical.

Regarding potential maternal toxicity, all therapies for breast cancer carry risks that can significantly impact patients in the short and long term and these risks must be weighed against the short- and long-term benefits. Some of these considerations are similar to risks in the general population, but may be of more concern for pregnant patients. For example, in general, anthracycline-based chemotherapy can lead to heart failure and decreased left ventricular ejection fraction (LVEF) especially at higher doses. While current guidelines recommend all patients receive standard monitoring of LVEF with regular cardiac imaging, particular attention may be considered for pregnant patients including consideration of a maternal echocardiogram prior to initiation of anthracycline-based chemotherapy and a fetal echocardiogram to ensure normal cardiac anatomy [48]. Anthracycline use has also been linked to an increased risk of developing secondary hematologic malignancies, mainly acute myeloid leukemia [49]. Taxanes, such as paclitaxel and docetaxel, are associated with neuropathy, fluid retention, and gastrointestinal reflux, which may prove challenging as pregnancy itself can also be associated with these effects. Most chemotherapies for early breast cancer are associated with significant risk of alopecia which can be distressing for many women. While, in general, more women are electing to pursue scalp cooling for prevention of alopecia during chemotherapy for early breast cancer, this modality has not been studied for use in gestational breast cancer. However, there is a case report that suggests possible safety [50, 51]. Risks and benefits of scalp cooling may be discussed with patients.

### Metastatic breast cancer

Metastatic breast cancer during pregnancy is rare. Incidence approaches up to 5% in patients with previously diagnosed early-stage breast cancer [52]. The most common sites of metastasis for gestational breast cancers are similar to those in the non-pregnant population and include bone, lung, and liver [53]. In general, many of the same treatment principles hold safety of the mother and fetus must be balanced when planning delivery, and radiation is contraindicated so palliative radiation should be delayed until the postpartum setting [54]. Importantly, there are no reported cases of metastatic disease of the breast to the fetus, although there are reports of placental metastasis. All placental tissue should be examined by an experienced pathologist following delivery.

Overall, treatment regimens for pregnant patients with breast cancer should closely follow the guidelines for non-pregnant patients, with adjustments to minimize any fetal risks. A multidisciplinary (high-risk obstetric, oncological, pediatric, genetic) team should therefore be established upon diagnosis to longitudinally manage the complexities of treatment and ensure the best possible outcomes for both the mother and the fetus.

### Treatment considerations by pregnancy trimester

For patients presenting in the *first trimester* with advanced stage breast cancer and high-risk early-stage breast cancer, it is imperative that physicians discuss the risks and benefits of treatment with or without pregnancy. Some patients may make the very challenging decision to terminate a desired pregnancy and pursue treatment that is critical to their well-being. While understudied, it is unclear whether pregnancy termination confers improvements in breast cancer outcomes and survival. In patients who choose to continue with their pregnancy, oncologic treatment must be initiated promptly, as a delay may lead to a worse maternal prognosis. Prior to initiating any treatment, a fetal ultrasound is conducted to rule out any pre-existing fetal abnormalities [55]. Chemotherapy is associated with a high risk of malformations if administered during this trimester and should therefore be avoided. However, during the first trimester, patients may choose to undergo surgical resection if critical. Discussion regarding the risks and benefits of anesthesia during the first trimester is best conducted in a multidisciplinary fashion with the breast surgeon and high-risk obstetrician.

Chemotherapy can generally be administered safely during the *second trimester*, as most teratogenic effects occur earlier in gestation. Treatment initiated after the first trimester reduces the highest risks of miscarriage and fetal malformation. Chemotherapy during this period is associated with a 3% risk of congenital malformations, which is comparable to the general population risk in the USA [56]. Standard adjuvant and neoadjuvant chemotherapy regimens consisting of anthracycline, cyclophosphamide, and taxane combinations are recommended, with adjustments based on the patient’s condition and tumor subtype. Anthracycline-based regimens continue to be preferred due to their efficacy in breast cancer treatment and long history of relative safety during pregnancy [57]. However, a recent 2024 study found adding taxanes to anthracycline regimens is also safe and does not result in less favorable obstetric and fetal outcomes [35]. If surgery is needed during pregnancy, it is preferred that patients undergo surgery early during this trimester.

In the *third trimester*, chemotherapy may be started and/or continued. Given the general association of chemotherapy and other breast cancer treatments with increased maternal and fetal complications [6], it is recommended that patients receive biweekly fetal monitoring. Delivery should be coordinated with the patient’s obstetrics team and planned as close to term as possible, ideally allowing for a 3-week interval from the last chemotherapy administration to promote maternal bone marrow recovery and reduce the risk of hematologic toxicity [58]. The mode of delivery is determined by obstetric indications. A vaginal delivery is the preferred method of delivery and oncologic treatment may be continued immediately after vaginal delivery. A cesarean delivery is only considered for obstetric indications, such as fetal breech presentation or intolerance to labor, and it is recommended that oncologic treatment be resumed 1 week after surgery to allow for appropriate wound healing.

## Maternal and fetal outcomes

### Maternal outcomes

The diagnosis of breast cancer during pregnancy is often delayed due to the physiologic changes of pregnancy and lactation. The increased breast density, nodularity, and heightened vascularity in pregnancy can obscure the early signs and symptoms of cancer and may initially be attributed to other pregnancy-related conditions [12]. Additionally, pregnant women with breast cancer are more likely than non-pregnant women to have aggressive tumor subtypes, such as triple-negative or HER2-positive cancers [59, 60]. These known risk factors (including delay in diagnosis and aggressive tumor biology) are associated with more advanced stage breast cancers and overall worse breast cancer outcomes. A 2022 study found that patients diagnosed with PABC in the first trimester had an overall survival rate of 81.3%, compared to 60.0 and 64.9% for those diagnosed in the second and third trimesters, respectively [61]. A 2016 meta-analysis found that patients with pregnancy-associated breast cancer had decreased disease-free survival compared to the control population [62]. However, other existing research suggests that pregnancy itself does not necessarily exacerbate cancer outcomes. A cohort study comparing 311 pregnant patients with 865 non-pregnant patients with breast cancer found that pregnancy did not adversely impact disease-free or overall survival [55]. Additionally, research evaluating 75 pregnant women treated with chemotherapy regimens, including 5-fluorouracil, doxorubicin, and cyclophosphamide, showed statistically significant improvements in survival and prognosis compared to non-pregnant women [63]. This conflicting data highlights the importance of developing future prospective studies to better understand and improve outcomes for patients with gestational breast cancer.

Coping with a cancer diagnosis and treatment during pregnancy can be a highly emotional and vulnerable experience. This may have a dramatic impact on a woman’s psychological well-being, complicating her ability to manage the disease, its treatment course, and her prenatal care. A 2011 study indicates that women who experience obstetric challenges following cancer treatment such as cesarean delivery, preterm birth, or were advised to terminate their pregnancy without subsequent fertility support are at increased risk for long-term distress [64]. Patients’ experiences of the ups and downs of young motherhood all while managing fear of cancer recurrence is challenging. Referral to a reproductive or oncologic psychiatrist can therefore be beneficial in managing these complex emotions and dual roles as patients and caregivers. Cognitive behavioral therapy is recommended as the gold standard in psychosocial treatment and can provide crucial support during this complex experience [65]. Psychological counseling should focus on addressing prenatal and postpartum emotions and providing women with safe and healthy coping strategies when in stressful or difficult situations. The desire to breastfeed may also impact psychological health during this time. Existing data suggest that breast cancer survivors who attempt breastfeeding can safely breastfeed [66]. Referral to a lactation consultant may be considered to improve this experience for affected women [33].

Strong social support is an important component of overall well-being during pregnancy and is even more critical for women diagnosed with gestational breast cancer. Poor familial support can lead to increased prenatal anxiety and depression, while a supportive partner and community can help alleviate and buffer symptoms of depression during pregnancy and postpartum. Patients with gestational breast cancer may be further supported by social workers who can help identify patients’ needs and recommend the appropriate resources and services. These referrals may include individual or group counseling and support aimed at fostering a strong support network and empowering women to be an active participant in their care.

### Fetal/childhood outcomes

Most childhood outcomes following treatment of gestational breast cancer are generally favorable. Nevertheless, children of mothers who received breast cancer treatment during pregnancy are appropriately carefully monitored after birth for any adverse effects or complications. We recommend obtaining a complete blood count, liver panel, and renal function assessment within a few days of birth to evaluate for cytopenias and other renal and hepatic toxicities from in utero treatment exposures. Children are at higher risk of these complications if born less than three weeks after the mother’s final chemotherapy cycle. Infants exposed to platinum-based chemotherapy in utero should undergo auditory screening within the first year and again at age five to detect any potential hearing impairments. For those exposed to anthracyclines, a cardiac echocardiogram is recommended within the first year of life, followed by routine evaluations every three years to monitor for cardiotoxicity [67].

Apart from these preliminary tests, children born to mothers with gestational breast cancer should be followed long term for secondary malignancies and neurodevelopmental disorders. According to an existing study of 129 children born to mothers with pregnancy-associated breast cancer, there were no statistically significant differences in birth weight or cognitive development compared to the general population [68]. While current literature does not show an elevated risk of cancer in children exposed to chemotherapy in utero, there are theoretical risks based on patterns observed in childhood cancer survivors. In a 2020 study of 132 six-year olds born to mothers diagnosed with cancer during pregnancy, there was an average decrease of 6 points in verbal IQ compared to their peers as well as lower visuospatial long-term memory, although other cognitive functions were normal [69]. Overall, while most outcomes for these children are within expected ranges, ongoing surveillance is needed to address potential long-term effects and initiate intervention in a timely manner if needed.

### Termination in gestational cancer

Diagnosing breast cancer early in pregnancy involves a particularly sensitive and challenging scenario where the option of pregnancy termination may be discussed when legal. For the patient, this decision involves a combination of medical and personal factors. For the oncologist, this requires a careful approach to counseling, as there is a potential for severe maternal outcomes if oncologic treatment is delayed, yet pregnancies are often highly desired. Given the urgency of the situation and the impact of gestational age in the feasibility of termination of pregnancy, it is crucial to conduct the diagnosis and workup as quickly as possible and involve the obstetric team to aid in counseling. Physicians must lead and support patients through a shared decision-making process that balances their values with the medical implications of continuing or terminating the pregnancy. This involves a thorough discussion of all available options and alternatives where the potential risks and benefits to both maternal and fetal health are communicated and carefully considered.

### Adjuvant surveillance during pregnancy

Many women diagnosed with breast cancer during pregnancy may desire future pregnancies, especially with advancing maternal age and increasing incidence of breast cancer in younger women. As a result, oncofertility counseling and treatment has become an increasingly important component to providing comprehensive care for premenopausal women with breast cancer. This is further supported by a 2021 meta-analysis which revealed that women who underwent breast cancer treatment had a 60% reduced likelihood of achieving future pregnancies compared to the general population [70]. Fortunately, the same study indicated that those who did achieve pregnancy after breast cancer did not experience a higher rate of adverse fetal outcomes or reproductive complications. A cohort study led by our group studied 75 women with pregnancy-associated breast cancer, including 25 women with gestational breast cancer. Although this is a small cohort, the data show similar survival outcomes when comparing women with subsequent pregnancies to women without subsequent pregnancies. Importantly, recent evidence from the POSITIVE trial has established the relative safety of interrupting endocrine therapy to pursue pregnancy after diagnosis of hormone receptor-positive breast cancer [52]. As a result of these increasing safety data, it is possible that more patients will pursue pregnancy after early breast cancer and would be observed by medical oncologists during their pregnancies.

For patients treated for early-stage breast cancer, there is no current, established standard for monitoring for breast cancer recurrence during pregnancy. Some providers will elect to pursue systemic staging scans prior to discontinuation of endocrine therapy for pregnancy, although the benefit of this surveillance has not been established by clinical trial evidence. Additionally, some providers elect to utilize liquid biopsy, plasma testing for circulating tumor DNA (ctDNA), to monitor for breast cancer recurrence before or during pregnancy in breast cancer survivors. ctDNA detection has been strongly associated with breast cancer recurrence in the non-gestational setting [71]. A 2022 case study found that ctDNA testing detected a local breast cancer recurrence in a patient at 33-week gestation, before radiological detection [72]. At the time of publication, serial ctDNA monitoring was negative after this patient underwent resection, and she delivered a healthy baby. Additionally, ctDNA has identified distant metastatic recurrence in gestational breast cancer. Further data will be needed to determine whether early detection of recurrence during pregnancy via ctDNA improves outcomes for gestational breast cancer recurrences.

## Conclusion

The management of gestational breast cancer involves adapting standard oncologic treatment regimens while making necessary modifications to preserve maternal and fetal safety. Despite ongoing research and clinical advances that enhance our understanding of breast cancer during pregnancy, significant knowledge gaps remain. Further research is needed to evaluate the safety profiles of various diagnostic modalities, surgical interventions, chemotherapeutics, and targeted systemic therapies for both maternal and fetal health. Given the increasing use of targeted therapies for breast cancer treatment, including immune checkpoint inhibitors, PARP inhibitors, and CDK 4/6 inhibitors, continued research will be essential for understanding the utility of these agents in the management of gestational breast cancer. As incidence of gestational breast cancer increases, it is imperative to prioritize ongoing research and to encourage collaborative medical and psychological care when treating this important population.

## Data Availability

No datasets were generated or analysed during the current study.
